# Proof of concept for eradication of vancomycin resistant *Enterococcus faecium* from broiler farms

**DOI:** 10.1186/1751-0147-55-46

**Published:** 2013-06-11

**Authors:** Oskar Nilsson, Ivar Vågsholm, Björn Bengtsson

**Affiliations:** 1 Department of Animal Health and Antimicrobial Strategies, National Veterinary Institute, SE, Uppsala 751 89, Sweden; 2 Department of Clinical Sciences, Swedish University of Agricultural Sciences, SE, Uppsala 756 51, Sweden; 3 Department of Biomedical Sciences and Veterinary Public Health, Swedish University of Agricultural Sciences, Uppsala, Sweden

**Keywords:** VRE, Eradication, Proof of concept, Disinfection

## Abstract

**Background:**

Vancomycin resistant enterococci (VRE) in Swedish broiler production has been shown to persist at farms between batches. The aim of this study was therefore to determine the possibility to eliminate VRE by disinfection of compartments in broiler houses as a proof of concept.

**Findings:**

VRE could not be detected in environmental samples from the disinfected test compartments in the broiler houses but was detected in environmental samples from the control compartments. The proportion of broilers colonized with VRE decreased in both the test and the control compartments.

**Conclusions:**

The results are promising and show that the occurrence of VRE in broiler houses can be reduced by adequate cleaning and disinfection with a combination of steam and formaldehyde.

## Findings

Among randomly selected enterococci from broilers in Sweden, the proportion of vancomycin resistant enterococci (VRE) is very low [1]. However, the prevalence of broilers colonized with VRE increased rapidly from 2000 to 2005 due to the spread of one major clone of *vanA* carrying *Enterococcus faecium*[2]. This clone has been found to persist at farms between batches [3]. There appears to be no constant introduction of VRE, neither from the hatcheries nor from the feed [4]. Together this indicates that the prevalence of broilers colonized with VRE would decrease if VRE could be eliminated from the farms. No clear differences in management routines between Swedish farms contaminated and not contaminated with VRE have been found [4]. This could indicate that none of the cleaning and disinfection routines normally used is sufficiently effective. The aim of this study was therefore to, as a proof of concept, determine the possibility to eliminate VRE from compartments in broiler houses by a new disinfection routine.

Two farms, in southern Sweden, with a history of broilers colonized with VRE were chosen and the houses were either cleaned and disinfected according to the farm’s normal routines (control compartments) or cleaned according to the farm’s normal routines and disinfected with a combination of steam and formaldehyde (test compartments), as outlined by Gradel *et al.*[5]. The efficacy towards enterococci of the disinfection method chosen for the intervention has been shown under both laboratory and field conditions [5,6]. The farms normal cleaning and disinfection routines included pressure wash and disinfection with a glutaraldehyde (Farm A) or a chloride compound (Farm B). At Farm A; two compartments in one house were used as test compartments and disinfected at a temperature of 50°C for 4 h and two compartments in an identical house were used as control. At Farm B; a single compartment house was used as test compartment and disinfected at a temperature of 60°C for 4 h whereas two compartments in one house (each of the same size as the test compartment) were used as controls. The size of the test compartments were approximately 1900 square meters (5700 cubic meters) at Farm A and 1600 square meters (4800 cubic meters) at Farm B. At both farms, 90 g of Formalin (23%) per cubic meter were used.

From each farm, caecal samples from ten birds per house or compartment from one (Farm A) or two (Farm B) batches of broilers sent for slaughter preceding the intervention and two batches of broilers following the intervention were analyzed for the presence of VRE as previously described (Table 1) [2,3]. Briefly, caecal content (0.5 grams) was suspended in 4.5 mL from which 0.1 mL was streaked on Slanetz–Bartley agar (Oxoid, Basingstoke, UK) supplemented with vancomycin (16 mg/L; Sigma-Aldrich, Steinheim, Germany) and incubated at 37°C for 48 h. Caecal samples from broilers raised at Farm A could only be identified by house and not by compartment and therefore all samples were grouped accordingly. None of the batches sampled were treated with antibiotics. In addition, boxes in which the chicks for the batch directly following the disinfection were delivered were sampled and cultured in the same way as the environmental samples (see below). At Farm A, ten boxes from each house were sampled whereas at Farm B only nine boxes in total were sampled since the day old chicks for both test and control compartments were of the same consignment. Environmental samples (air inlet, feed line and water line) from each compartment were collected before and after disinfection. All environmental samples were taken in duplicate except samples from the water line after disinfection at Farm A. Environmental samples were collected with sterile cloths and cultured for qualitative detection of VRE as previously described [3]. Briefly, Enterococcosel (Merck, Darmstadt, Germany) was added and the samples were treated in a stomacher before 0.1 mL was streaked on Slanetz–Bartley agar (Oxoid) supplemented with vancomycin (16 mg/L; Sigma-Aldrich) both directly and after incubation in 37°C for 3–4 hours. From each sampling occasion, one isolate was selected at random for species identification and susceptibility testing with VetMIC E-cocci (SVA, Uppsala, Sweden) as previously described [2,7].

**Table 1:** Proportion of caecal and environmental samples positive for vancomycin resistant enterococci (VRE) (number positive/number cultured)

	**Farm A**		**Farm B**	
	**Test compartments**	**Control compartments**	**Test compartment**	**Control compartments**
Caecas two batches before disinfection	na	na	1/10	5/20
Caecas one batch before disinfection	4/10	3/10	2/10	8/20
Environmental samples before disinfection	10/12	6/12	6/6	4/12
Environmental samples after disinfection	0/10	2/10	0/6	6/12
Caecas one batch after disinfection	1/10	0/10	0/10	1/20*
Caecas two batches after disinfection	0/10	2/10	0/10	1/20*

At Farm A the temperature in the test compartment was raised to 50°C for 4 hours and at Farm B to 60°C. At both farms the control compartment(s) were cleaned and disinfected according to the respective farms normal routines.

na = samples not taken.

* Both the caecas positive for VRE were from broilers raised in one of the control compartments whereas all caecas from both batches from the other control compartment were negative for VRE.

The overall aim of this study was to, as a proof of concept, show that there are ways to decrease the level of VRE contamination in the environment at broiler farms and thereby also lower the prevalence of broilers colonized with VRE at slaughter. Both temperatures tested in the proof of concept study reduced the amount of VRE after disinfection below the detection limit of the methodology for environmental samples (Table 1). In contrast, VRE were still isolated after disinfection by the farms’ normal routines. With respect to presence of VRE in the caecas of the birds, the study was not conclusive since the proportion of chickens colonized with VRE diminished also in the control compartments (Table 1). Notably, at Farm A VRE was isolated from one sample from the first batch of broilers raised in the test compartments after disinfection but in no sample from the second batch. Furthermore, VRE was not isolated in samples from the first batch raised in the control compartments after disinfection, but from one sample from the second batch. These inconsistencies could be due to lack of sensitivity in the sampling and culturing procedure. However, cross contamination from other houses at the farm is another plausible explanation. Similar inconsistencies were also experienced by Gradel *et al.* when developing the disinfection method [5,6]. The birds were probably not colonized when they arrived at the farms since VRE was not isolated from any of the samples taken from the boxes in which the chicks were delivered. All isolates chosen for species identification and susceptibility testing were *E. faecium* and the MIC of vancomycin for these isolates was >128 mg/L.

It has been suggested that it will take decades for VRE to disappear from farms even without the selective pressure of glycopeptides [8]. No interventions were however included in that prediction. Our study shows that the VRE persisting in the broiler houses can be reduced and perhaps even eliminated by disinfection with a combination of steam and formaldehyde. A reduction of VRE contamination in the broiler houses could lead to fewer birds colonized and consequently to further reduction of VRE occurrence in the houses by the next cleaning and disinfection. If so, with maintained bio-security and appropriate cleaning and disinfection after each batch, the occurrence of VRE in Swedish broiler production would decrease and eventually this could lead to eradication. Furthermore, the effectiveness of the tested disinfection method against VRE indicates that it can also be used for other species since enterococci are known to be robust bacteria [9].

In conclusion, the results are promising and suggest that disinfection with a combination of steam and formaldehyde was more efficient in reducing the level of VRE contamination than the farms’ normal disinfection routines. Hence, the occurrence of VRE can probably be reduced by adequate methods for cleaning and disinfection. Consequently, a continuous reduction could lead to eradication of VRE from the Swedish broiler industry. Further work to elucidate the possibility to control VRE in Swedish broiler production could include large scale disinfection studies where all of the houses at farms are disinfected.

## Competing interest

The authors declare that they have no competing interests exist.

## Authors’ contribution

The study was designed by all authors. ON did the field work and the laboratory work. ON drafted the manuscript and all authors revised, read and approved the final manuscript.
